# Supplementary material for: Young Patients with Anorexia Nervosa: The Contribution of Post-Traumatic Stress Disorder and Traumatic Events
Source: Medicina (Kaunas). 2020 Dec 22;57(1):2. doi: 10.3390/medicina57010002 (PMC7822187; doi:10.3390/medicina57010002)
Supplement: Supplementary file 1 [file medicina-57-00002-s001.pdf]

Supplementary tables

Table S1. differences between patients with and without childhood trauma.

	Childhood trauma (n= 51)	Non-childhood trauma (n= 12)		
	Mean (SD)	Mean (SD)	z	p
**Years of illness**	3.35 (2.54)	3.50 (3.45)	-.267	.789
**Illness onset**	16.80 (2.40)	16.58 (2.64)	-.736	.462
**Bmi**	15.40 (2.73)	13.99 (1.74)	-1.839	.066
**N of hospitalizations**	1.92 (1.40)	2.17 (1.85)	-.369	.712
**EDE-Q**				
**Restrain**	2.96 (1.89)	1.87 (2.15)	-1.706	.088
**Food concern**	3.09 (1.43)	2.52 (1.83)	-1.323	.186
**Shape concern**	3.89 (1.62)	3.23 (1.64)	-1.200	.230
**Weight concern**	3.47 (1.69)	2.65 (1.85)	-1.336	.182
**Total score**	3.35 (1.55)	2.50 (1.61)	-1.488	.187
**BDI**	17.74 (7.83)	13.25 (8.67)	-1.980	**.048**
**STAI-State**	58.10 (11.64)	51.00 (14.44)	-1.563	.118
**State-Trait**	58.68 (16.85)	52.58 (12.48)	-1.840	.066
**DES**	24.57 (17.58)	13.18 (12.32)	-2.343	**.019**

BMI = body mass index; EDE-Q = eating disorder examination questionnaire; BDI = Beck depression inventory; STAI = State-trait anxiety inventory; DES = dissociative experiences scale.

Table S2. Differences between patients with trauma before illness onset and patients with trauma after Illness onset.

	Trauma before illness onset (n= 40)	Trauma after illness onset (n= 12)		
	Mean (SD)	Mean (SD)	z	p
**Years of illness**	3.42 (2.85)	3.42 (2.35)	-.320	.749
**Illness onset**	16.85 (2.30)	16.83 (2.66)	-.198	.843
**Bmi**	15.19 (2.68)	14.53 (1.67)	-.652	.515
**N of hospitalizations**	2.08 (1.47)	1.92 (1.56)	-.509	.611
**EDE-Q**				
**Restrain**	2.73 (2.09)	2.74 (2.85)	-.296	.767
**Food concern**	2.71 (1.56)	3.38 (1.86)	-1.153	.249
**Shape concern**	3.83 (1.55)	3.89 (1.87)	-.263	.813
**Weight concern**	3.21 (1.90)	3.64 (1.97)	-.771	.441
**Total score**	3.12 (1.66)	3.32 (1.68)	-.266	.790
**BDI**	15.30 (7.60)	17.50 (9.20)	-.633	.527
**STAI-State**	55.32 (13.25)	60.60 (13.07)	-1.009	.313
**State-Trait**	55.00 (17.94)	60.40 (13.01)	-.785	.433
**DES**	21.05 (16.56)	21.13 (15.41)	-.033	.974

BMI = body mass index; EDE-Q = eating disorder examination questionnaire; BDI = Beck depression inventory; STAI = State-trait anxiety inventory; DES = dissociative experiences scale.

Table S3. differences between patients with and without multiple traumas.

	multiple traumas (n= 38)	Non-multiple traumas (n= 23)		
	**Mean (SD)**	**Mean (SD)**	**z**	**p**
**Years of illness**	3.61 (2.87)	3.13 (2.51)	-.607	.544
**Illness onset**	16.61 (2.14)	16.83 (2.84)	-.362	.718
**Bmi**	15.73 (2.75)	14.27 (2.23)	-2.337	**.019**
**N of hospitalizations**	1.87 (1.38)	2.13 (1.69)	-.264	.791
**EDE-Q**				
**Restrain**	2.81 (2.16)	2.52 (1.83)	-.716	.474
**Food concern**	2.89 (1.52)	3.04 (1.62)	-.313	.754
**Shape concern**	4.05 (1.59)	3.29 (1.76)	-1.516	.130
**Weight concern**	3.33 (1.74)	3.14 (1.96)	-.265	.791
**Total score**	3.27 (1.61)	2.96 (1.61)	-.645	.519
**BDI**	16.88 (7.77)	16.32 (8.99)	-.086	.932
**STAI-State**	58.10 (11.35)	53.68 (13.99)	-1.307	.191
**State-Trait**	56.40 (18.95)	58.50 (13.30)	-.222	.824
**DES**	23.63 (18.90)	18.49 (13.28)	-.912	.362

BMI = body mass index; EDE-Q = eating disorder examination questionnaire; BDI = Beck depression inventory; STAI = State-trait anxiety inventory; DES = dissociative experiences scale.

Table S4. Differences between patients with and without psychiatric familiarity.

	Psychiatric familiarity (n= 24)	Non- Psychiatric familiarity (n= 34)		
	**Mean (SD)**	**Mean (SD)**	**z**	**p**
**Years of illness**	3.16 (2.74)	3.76 (2.84)	-1.084	.278
**Illness onset**	16.79 (2.75)	16.71 (2.37)	-.176	.861
**Bmi**	14.60 (2.13)	15.53 (2.76)	-1.153	.249
**N of hospitalizations**	2.38 (1.76)	1.82 (1.29)	-.894	.371
**EDE-Q**				
**Restrain**	2.84 (2.04)	2.61 (2.03)	-.522	.602
**Food concern**	2.75 (1.69)	3.07 (1.50)	-.749	.454
**Shape concern**	3.65 (1.70)	3.78 (1.70)	-.426	.670
**Weight concern**	3.32 (1.73)	3.24 (1.97)	-.010	.992
**Total score**	3.11 (1.61)	3.18 (1.67)	-.488	.625
**BDI**	15.71 (8.69)	17.02 (8.17)	-.415	.678
**STAI-State**	56.41 (9.91)	57 (14.69)	-.332	.740
**State-Trait**	58.86 (11.42)	57.50 (16.29)	-.010	.992
**DES**	25.54 (21.37)	20.02 (14.36)	-.675	.500

BMI = body mass index; EDE-Q = eating disorder examination questionnaire; BDI = Beck depression inventory; STAI = State-trait anxiety inventory; DES = dissociative experiences scale.

Table S5. Differences between patients with and without EDs familiarity.

	EDs familiarity (n= 10)	Non- EDs familiarity (n= 50)		
	Mean (SD)	Mean (SD)	z	p
**Years of illness**	3.68 (3.01)	3.46 (2.62)	-.060	.952
**Illness onset**	17.00 (3.71)	16.72 (2.21)	-.441	.659
**Bmi**	15.38 (2.54)	15.12 (2.60)	-.704	.481
**N of hospitalizations**	3.50 (2.07)	1.72 (1.18)	-2.969	**.003**
**EDE-Q**				
**Restrain**	2.91 (2.09)	2.60 (2.98)	-.254	.800
**Food concern**	3.24 (2.05)	2.87 (2.44)	-.683	.495
**Shape concern**	4.14 (1.51)	3.64 (1.68)	-.746	.456
**Weight concern**	3.87 (1.55)	3.11 (1.86)	-1.139	.255
**Total score**	3.46 (1.63)	3.06 (1.60)	-.593	.553
**BDI**	20.83 (9.67)	15.79 (7.75)	-1.559	.119
**STAI-State**	61.60 (8.45)	55.26 (13.26)	-1.279	.201
**State-Trait**	61.40 (8.14)	56.21 (17.52)	-.569	.569
**DES**	22.68 (14.12)	21.76 (18.16)	-.618	.537

BMI = body mass index; EDE-Q = eating disorder examination questionnaire; BDI = Beck depression inventory; STAI = State-trait anxiety inventory; DES = dissociative experiences scale.

Table S6. Differences in categorical variables between patients with and without risk factors.

	Chidlhood trauma	Non-childhood trauma	
	N (%)	N(%)	Fisher's test (p)
**RAN**	31 (60.8)	12 (39.2)	**.007**
**BPAN**	20 (100)	0 (0)	
**SH**	9 (17.6)	1 (8.3)	.671
**SA**	10 (19.6)	0 (0)	.186
	**Trauma before illness onset**	**Trauma after illness onset**	
	**N (%)**	**N(%)**	**Fisher's test (p)**
**RAN**	24 (70.6)	10 (29.4)	.179
**BPAN**	16 (88.9)	2 (11.1)	
**SH**	5 (55.6)	4 (44.4)	.185
**SA**	7 (87.5)	1 (8.3)	.663
	**Multiple traumas**	**Non-multiple traumas**	
	**N (%)**	**N(%)**	**Fisher's test (p)**
**RAN**	22 (53.7)	19 (46.3)	.055
**BPAN**	16 (80)	4 (20)	
**SH**	6 (66.7)	3 (33.3)	1.000
**SA**	7 (70)	3 (30)	.729
	**Psychiatric familiarity**	**Non-psychiatric familiarity**	
	**N (%)**	**N(%)**	**Fisher's test (p)**
**RAN**	18 (46.2)	21 (53.8)	.397
**BPAN**	6 (31.6)	13 (68.4)	
**SH**	4 (44.4)	5 (55.6)	1.000
**SA**	5 (50)	5 (50)	.726
	**EDs familiarity**	**Non-EDs familiarity**	
	**N (%)**	**N(%)**	**Fisher's test (p)**
**RAN**	8 (20)	32 (80)	.471
**BPAN**	2 (10)	18 (90)	
**SH**	3 (33.3)	6 (66.7)	.163
**SA**	1 (10)	9 (90)	1.000

RAN = restricter anorexia nervosa; BPAN = binge-purging anorexia nervosa; SH = self-harm; SA = suicide attempts; EDs = eating disorders.
